# Pediatric gallbladder torsion managed by laparoscopic cholecystectomy: a case report and scoping review

**DOI:** 10.3389/fped.2024.1506506

**Published:** 2025-01-14

**Authors:** Yi Sun, Zheng Fang, Xu Cao, Ting Zhang, Xiaobo Liu, Jie Zhang, Qianwei Xiong, Bin Wu, Xiangming Yan

**Affiliations:** ^1^ Department of General Surgery, Children’s Hospital of Soochow University, Suzhou, Jiangsu, China; ^2^ Department of Urology, Children’s Hospital of Soochow University, Suzhou, Jiangsu, China

**Keywords:** gallbladder torsion, diagnosis, laparoscopic cholecystectomy, child, treatment

## Abstract

Gallbladder torsion (GT), characterized by the axial rotation of the cystic duct and cystic artery, is a critical condition that predominantly affects elderly women and is infrequently observed in children. Chronic cholecystitis associated with incomplete GT is a particularly rare phenomenon. This article presents a pediatric case of chronic cholecystitis associated with incomplete GT. Ultrasonography and magnetic resonance cholangiopancreatography (MRCP) were utilized to confirm the diagnosis in the child. Laparoscopic cholecystectomy was successfully performed on the child, with no postoperative complications. For children with chronic cholecystitis associated with incomplete GT, clinical manifestations may improve. However, due to the presence of congenital anatomical abnormalities, children's symptoms may recur. Early laparoscopic cholecystectomy can prevent the acute onset of torsion and potential difficulties in diagnosis at later stages.

## 1 Introduction

GT, a rare emergency condition arising from the axial rotation of the cystic duct and cystic artery leading to gallbladder ischemia, was first described by Wendel A.V. in 1898 (1, 2). Since then, approximately 600 cases of GT in adults have been documented, predominantly in elderly women, with only 60 cases reported globally among children (3, 4). The diagnosis of GT in children poses a significant challenge to clinicians and radiologists due to its low incidence and nonspecific clinical manifestations, often resulting in delayed or ambiguous diagnoses and protracted disease courses. Following GT, the gallbladder is at risk of rapid necrosis, perforation, and biliary peritonitis. Timely diagnosis and surgical intervention are crucial for improving the prognosis of children with GT; the mortality rate associated with GT stands at 5%–6% (5, 6). Currently, no articles have reported on chronic cholecystitis associated with incomplete GT in children. This article presents a pediatric case of chronic cholecystitis associated with incomplete GT. The child underwent laparoscopic cholecystectomy at our institution in March 2024. Furthermore, the paper offers a comprehensive review of the literature on pediatric GT, summarizing the clinical manifestations, imaging characteristics, therapeutic approaches, and outcomes associated with pediatric GT.

## 2 Methods

English databases, including PubMed and Web of Science, were systematically searched to identify relevant literature. The search terms used were: “Torsion”, “Volvulus”, and “Gallbladder”. These terms were selected broadly to ensure the inclusion of all pertinent research and to minimize the risk of omitting any studies. The inclusion criteria were as follows: (1) Case reports specifically addressing GT; (2) Patients diagnosed with GT under the age of 18; (3) Case reports published after 1980. The exclusion criteria mainly included conference abstracts, non-English publications, inaccessible original texts, and studies lacking substantial information.

## 3 Results

**Figure 1:**
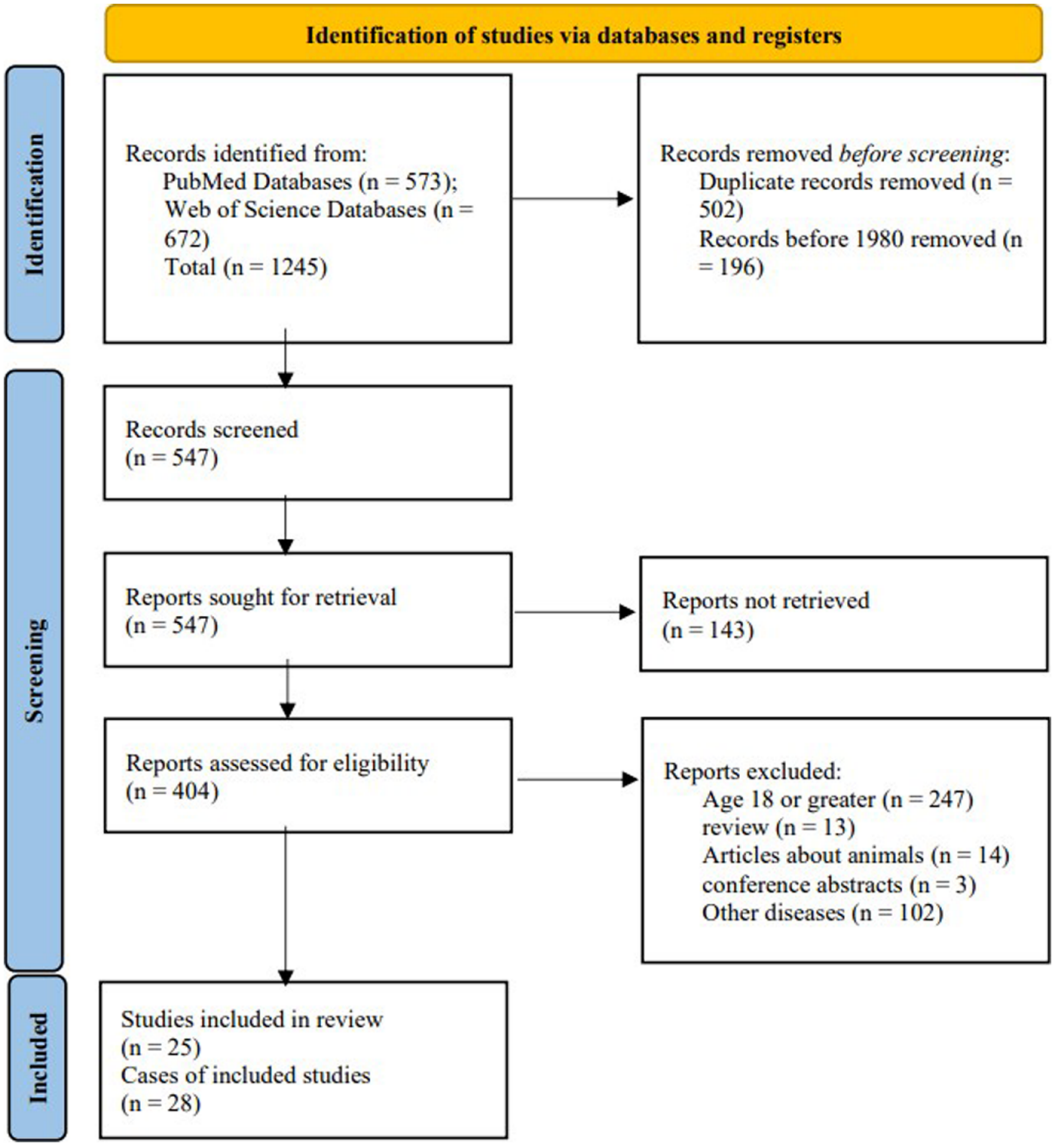
PRISMA flow diagram for our literature search.

**Figure 2:**
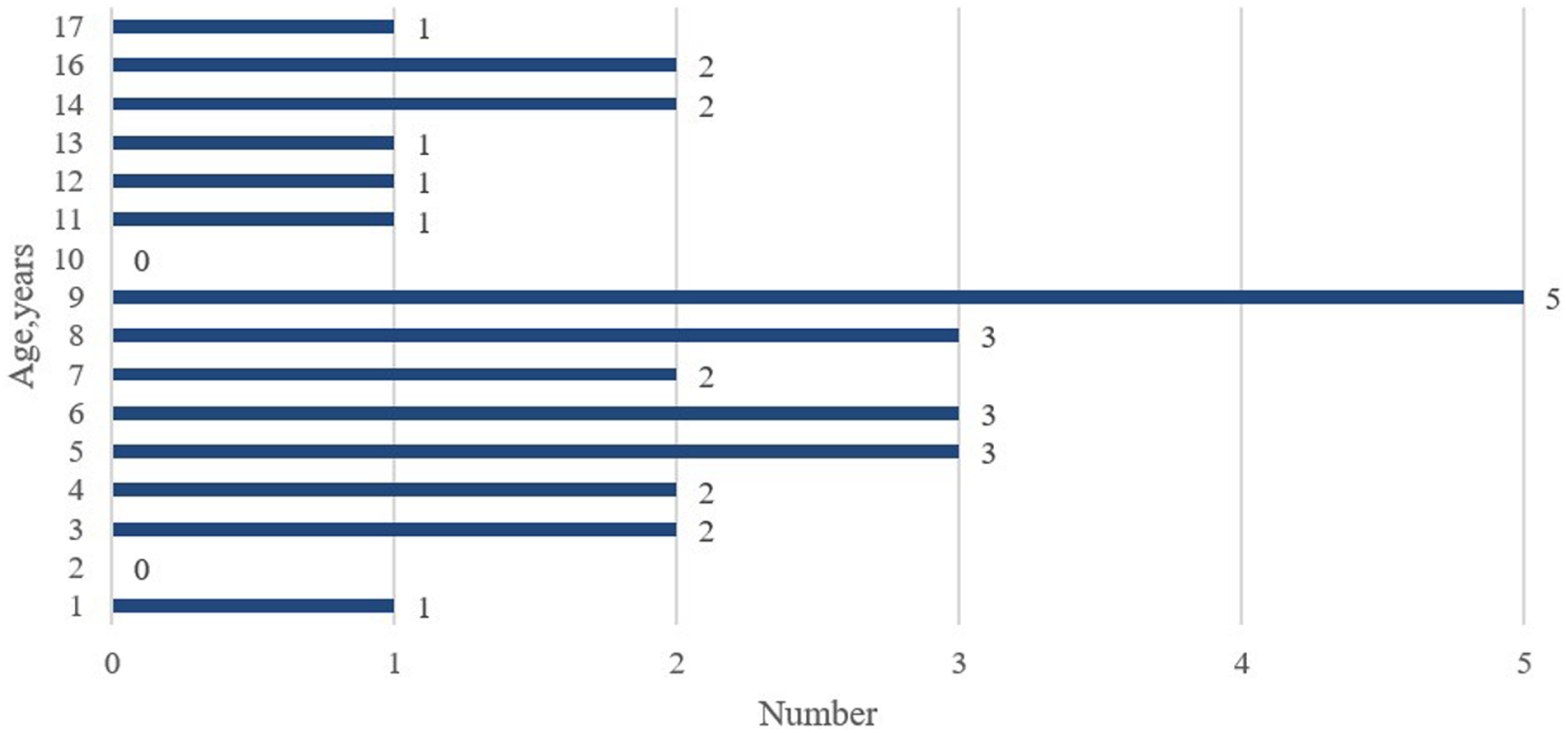
Age distribution of children with gallbladder torsion.

A total of 25 articles were included in this study, comprising 28 cases of pediatric GT (Figure 1). Including the case we reported, the total number of cases is 29. The male to female ratio was 2.63:1. The average age was 8.41 years, and the age distribution is illustrated in Figure 2. Children with GT presented with clinical manifestations such as vomiting, right upper abdominal pain, and low-grade fever. Physical examination primarily included tenderness in the right upper abdomen, abdominal muscle rigidity, and periumbilical tenderness. No children in this study presented with jaundice symptoms. Laboratory tests showed an elevated white blood cell count. Imaging characteristics included thickening of the gallbladder wall, gallbladder enlargement, ascites, and the figure-eight sign, among others. Intraoperative findings revealed 15 cases of cystic duct torsion, 8 cases of gallbladder neck torsion, and 2 cases of gallbladder body torsion. Except for 5 cases where the degree or angle of torsion was not explicitly mentioned, all other reported cases had a torsional angle of ≥180°. Two patients experienced prolonged disease duration, with the time from onset to surgery exceeding one week. The overall average length of hospital stay was 5 days, and no complications were observed during the follow-up period.

## 4 Case presentation

**Figure 3:**
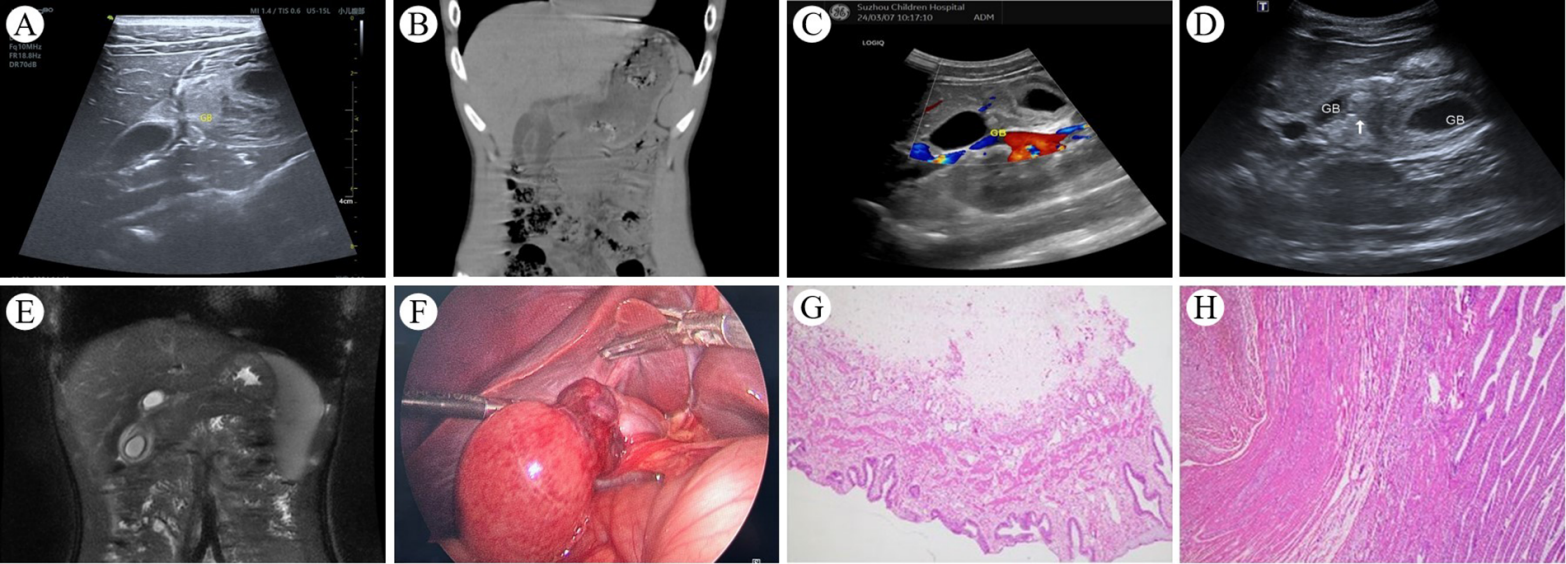
**(A)** Upon the child's first hospital admission, an abdominal ultrasound revealed a linear hypoechoic area in the body of the gallbladder, with thickening and edema of the walls at the body and base; **(B)** An abdominal CT scan showed a rough and thickened gallbladder wall, fluid accumulation in the gallbladder fossa, and a small amount of pelvic effusion; **(C)** abdominal ultrasound demonstrated a linear hypoechoic area in the body, with significant thickening of the walls at the body and base, and blood flow signals were detectable within the hypoechoic region. **(D)** During the child's second hospital admission, abdominal ultrasound showed a marked enlargement of the base and body of the gallbladder, with pronounced thickening of the walls, and a whirlpool-like hyperechoic area was observed between them; **(E)** Abdominal MRI combined with MRCP indicated an enlarged gallbladder, with a “figure of eight sign” at the middle part of the body, the gallbladder wall was significantly thickened and rough, and fluid was visible in the gallbladder fossa; **(F)** Intraoperative findings revealed a markedly enlarged gallbladder base, with significant edema and thickening of the wall, and torsion of the body. **(G** and **H)** chronic cholecystitis associated with GT.

A 14-year-old boy was hospitalized twice for persistent right upper quadrant abdominal pain. Upon the first admission, the primary complaint was “persistent right upper quadrant abdominal pain with nausea for more than 42 h”, without fever, vomiting, abdominal distension, or diarrhea. Intermittent right upper quadrant abdominal pain, present one month prior, had been temporarily relieved with omeprazole and pinaverium bromide. Physical examination revealed tenderness in the right upper quadrant of the abdomen. Complete blood count, C-reactive protein, biochemical tests, and serum amylase levels were within the normal range. Ultrasonographic findings indicated gallbladder enlargement, wall edema, and thickening, along with a linear hypoechoic region in the gallbladder body, indicative of GT or vascular compression (Figures 3A). Abdominal CT scan revealed a coarse and thickened gallbladder wall, associated with fluid accumulation in the gallbladder fossa and minimal pelvic effusion (Figure 3B). Following a period of fasting, fluid infusion, and antibiotic administration, abdominal pain was markedly reduced by the second hospital day. The patient displayed no adverse symptoms following the resumption of a regular diet. By the fourth day of hospitalization, complete blood count, C-reactive protein, biochemical tests, and serum amylase levels were within the normal range; however, the B-ultrasound continued to show a linear hypoechoic area within the gallbladder body, with detectable blood flow signals (Figure 3C). Given the lack of significant abnormalities in physical and laboratory examinations, the child was discharged. Within five days following discharge, the patient re-presented with persistent right upper quadrant abdominal pain. The clinical presentation was similar to the first admission, but the symptoms progressively worsened after readmission. Laboratory tests showed no significant abnormalities in complete blood count, C-reactive protein, liver function tests, and serum amylase. B-ultrasound revealed the gallbladder neck measuring approximately 24 mm × 13 mm with non-thickened walls, and the bottom and body measuring approximately 57 mm × 30 mm with significantly thickened walls, approximately 9 mm in thickness. Swirling hyperechoic areas were observed between the bottom and body, indicating GT (Figure 3D). MRCP showed an enlarged gallbladder, narrowing in the middle part of the body, and a figure-eight shape. The gallbladder wall was markedly thickened and rough, with fluid accumulation in the gallbladder fossa, indicating GT (Figure 3E). An emergency laparoscopic exploration was performed. Intraoperative exploration showed that the gallbladder neck and cystic duct are fixed by the mesentery, while the body of the gallbladder lacks mesenteric fixation to the inferior surface of the liver, leading to a free-floating condition within the gallbladder fossa (Figure 3F). An intraoperative diagnosis of “GT” was made, a laparoscopic cholecystectomy was performed to completely remove the gallbladder. The patient recovered well and was discharged on the fourth postoperative day. Post-surgical pathology revealed chronic cholecystitis associated with GT (Figures 3G,H). Follow-up abdominal ultrasound results were satisfactory.

## 5 Discussion

The etiology of GT in adults is multifactorial, encompassing: (1) Congenital anatomical anomalies, such as a floating gallbladder and gallbladder ptosis. (2) Age-related involution of visceral adipose tissue and laxity of associated ligaments (7), along with mesenteric expansion. (3) Atherosclerosis of the gallbladder arteries and morphological alterations of the cystic duct, which may act as fulcrums for torsion. (4) Intense physical activity and blunt abdominal trauma. In pediatric populations, congenital anatomical deformities predominate as the primary etiology of GT. The embryological foundation is rooted in the abnormal migration of the gallbladder during weeks 4 to 5 of gestation, leading to Gross Types A and B mesenteric anatomical variations, which predispose to a congenital floating gallbladder (8). GT is categorized as either complete (≥180°) or incomplete (<180°) (6). Complete torsion is associated with severe right upper quadrant colicky pain and gastrointestinal symptoms such as nausea and vomiting, attributable to severe ischemia. In contrast, incomplete torsion presents with mild symptoms and is frequently misdiagnosed as cholecystitis due to its subtle clinical presentation. This review summarizes the distribution of GT sites in 29 pediatric cases: 51.72% involve the cystic duct, 27.59% the gallbladder neck, and 6.89% the gallbladder body. The case presented in this paper, characterized by prompt symptom relief following initial admission, shares similarities with the case reported by Luo, P. et al. in 2014 (9). Notably, the ultrasound examination before the first discharge still indicated GT; however, blood flow signals were observed at the torsion site. Based on clinical manifestations and ultrasound findings, our case is indicative of incomplete GT. After the first discharge, increased activity and gastrointestinal motility in the child exacerbated the degree of GT, necessitating readmission. The torsion occurred at the body of the gallbladder. Among the 28 pediatric cases we reviewed, another case with torsion at the gallbladder body was reported by Inoue, S. et al. in 2011, involving a patient with intermittent mild abdominal pain (10). Both cases exhibited mild initial abdominal pain. Similar clinical features and the same site of torsion have also been reported in adults (11). Kwon et al. highlighted the rarity of GT at the body in their 2015 publication (12).

Accurate preoperative diagnosis of GT is challenging due to its rarity and similarities in clinical presentation and imaging findings to acute cholecystitis. Historical review reported a preoperative diagnosis rate of only 9% (13), which has recently increased to 26% according to more recent overviews (14). In our case report, the presence of clinical improvement during the first hospital admission further complicates achieving an accurate preoperative diagnosis of GT. Radiological examinations are central to diagnosing GT. Specific and nonspecific imaging findings of GT are presented in Table 1. MRCP is the most accurate diagnostic modality, with a 59% accuracy rate, outperforming ultrasonography (12%) and CT (30%) (36). Despite its lower accuracy, ultrasonography is crucial for diagnosing GT, as the lack of blood flow in the gallbladder wall, detected by CDFI, is a key diagnostic criterion (37) and the most observed specific feature in pediatric cases.

**Table 1:** Clinical data of 30 children with GT (1, 3, 5, 15–35).

	Total	
Gender		
Male	21	72.41%
Female	8	27.59%
Mean age, years (range)		
Male	9.00	(3, 16)
Female	6.88	(1, 17)
Overall	8.41	(1, 17)
Clinical manifestations		
Vomiting	22	75.86%
Abdominal pain	29	100.00%
Fever	16	55.17%
Physical examination		
Right upper abdominal tenderness	27	93.10%
Muscle rigidity	14	48.28%
Poor mental status	7	24.14%
Abdominal distension	4	13.79%
Diminished bowel sounds	2	6.89%
Periumbilical tenderness	2	6.89%
Laboratory tests		
Elevated white blood cells	22	73.33%
Elevated CRP	9	31.03%
Elevated liver enzymes	6	20.69%
Imaging features		
Nonspecific		
Asymmetric gallbladder wall thickening	23	79.31%
Gallbladder enlargement	19	65.51%
Ascites	14	48.28%
Gallbladder hydrops	3	10.34%
Biliary dilation	2	6.89%
Gallstones	0	0.00%
Specific		
Horizontal long axis of gallbladder	5	17.24%
No blood flow signal in gallbladder wall	7	24.14%
Figure of eight sign	2	6.89%
Whirl sign	1	3.45%
Beak sign at gallbladder neck	5	17.24%
Site of torsion		
Duct	15	51.72%
Neck	8	27.59%
Body	2	6.89%
Not mentioned	4	13.79%
Angle of torsion		
Less than 180°	0	0%
180°	6	20.69%
More than 180°	18	62.07%
Not mentioned	5	17.24%
Surgical methods		
Open cholecystectomy	15	51.72%
Laparoscopic cholecystectomy	14	48.28%
Course prolongation	2	6.89%
GT caused by abdominal trauma	2	6.89%

Data are presented as *n* (%), unless stated otherwise. Prolonged course is defined as the time from onset to surgery exceeding one week.

Cholecystectomy is the main treatment for pediatric GT. Given the gallbladder's relatively free-floating nature, the surgical procedure may not require the critical view of safety (CVS) techniques (15), simplifying the cholecystectomy process. Laparoscopic cholecystectomy is now the preferred method due to its minimal invasiveness and reduced surgical site infections. We intend to incorporate our pediatric GT case managed with laparoscopic cholecystectomy into the retrospective analysis by Uemura et al. on this procedure for pediatric GT (15), as shown in Table 2. Given the risks of recurrence and mechanical stress injuries (38, 39), we do not advocate gallbladder preservation. For children with chronic cholecystitis associated with incomplete GT, cholecystectomy is advocated to prevent symptom recurrence and serious complications from GT exacerbation. Early laparoscopic cholecystectomy is recommended for these cases.

**Table 2:** Cases of pediatric GT managed by laparoscopic cholecystectomy and with our patient added.

Author, year	Age (years)	Gender	Preoperative diagnosis	Time from onset to surgery (days)	Operation time (min)	Treatment for cystic duct	Complications	Postoperative hospital stay (days)
Kimura et al. (25)	11	M	GT	1	65	NA	None	4
Matsuda et al. (23)	7	F	GT	2	NA	NA	None	3
Inoue et al. (10)	9	M	GT	4	NA	NA	None	4
Farnsworth et al. (22)	6	M	Appendicitis	1	NA	NA	None	NA
Musthafa et al. (21)	17	F	biliary colic	1	NA	Endoloop	None	2
Uemura et al. (15)	3	M	GT	3	104	Endoloop	None	6
Hoshiet al. (20)	5	M	GT	NA	NA	NA	None	NA
	13	M	GT	NA	NA	NA	None	NA
Kruger et al. (19)	16	M	Appendicitis	2	NA	NA	None	2
Lemons et al. (18)	12	M	Cholecystitis	3	NA	Hem-o-lok® clips	None	1
Nuyts et al. (5)	1	F	GT	2	65	Hem-o-lok® clips	None	3
Ren et al. (3)	6	F	GT	2	95	Non-Absorbable Suture (4–0)	None	6
Tadesse et al. (17)	7	M	GT	3	NA	NA	None	4
Tiep et al. (16)	5	F	GT	1 month	NA	NA	None	7
Present case	13	M	Cholecystitis	11	99	Endoloop	None	4

F, female; M, male; NA, not available.

Patients with GT may experience complications such as gallbladder necrosis, perforation, biliary peritonitis, septic shock, and death. In some patients, GT is often accompanied by ascites, primarily caused by effusion from the twisted gallbladder. The effusion drains along the right colon to the lower right abdomen, causing pain in the lower right quadrant, which should be distinguished from acute appendicitis. Farnsworth et al. reported a case of GT misdiagnosed as acute appendicitis (22). The optimal time for surgical intervention in GT is within two days of symptom onset; delays beyond 2 days are associated with increased mortality (36). In adults with GT, the mortality rate is approximately 5%–6%, occurring exclusively in patients with delayed diagnosis. Therefore, early and accurate identification of GT is crucial (40). Among 29 pediatric cases of GT, there were no fatalities, and all reported cases were discharged within one week after cholecystectomy with fluid resuscitation and antibiotic administration.

We propose the following recommendations: (1) Although the clinical manifestations of GT are nonspecific, clinicians and radiologists should further enhance their ability to recognize this condition among children presenting with right upper quadrant abdominal pain to prevent diagnostic delays. (2) In children with chronic cholecystitis associated with incomplete GT, early laparoscopic cholecystectomy can prevent the serious complications associated with the exacerbation of GT. (3) MRCP is the most effective imaging method; when specific imaging findings are present, surgical exploration should be actively pursued to avoid missing the golden treatment window within two days of onset.

## Data Availability

The original contributions presented in the study are included in the article/Supplementary Material, further inquiries can be directed to the corresponding author/s.
